# 

**Published:** 1900-02-03

**Authors:** 


### NOTICE TO CORRESPONDENTS.

All MS., Letters, Books for Review, and other matters intended for the Editor should be addressed The Editor, "The Hospital," The Hospital Building, 28 & 29, Southampton Street, Strand, W.C.

The Editor cannot undertake to return rejected MS., even when accompanied by stamped directed envelope.

**Notice to Advertisers and Subscribers.**

All Advertisements, Orders for Copies of The Hospital, and Business Communications should be addressed to **THE MANAGER (Not to the Editor)**, The Hospital, The Hospital Building, 28 & 29, Southampton Street, Strand, London, W.C.

The Cost of Subscription, post free, is as follows:—Per week, 2½d.; per quarter, 2s. 8d.; per half-year, 5s. 3d.; per annum, 10s. 6d. Foreign:—Per annum, 15s. 2d., payable, in all cases, in advance.

**NOTICE.—Vol. XXVI. of "The Hospital" (April, 1899, to September, 1899), in handsome cloth binding, is now on sale at the Office of this Journal, price 7s. 6d. Cases for binding the Numbers contained in this Volume 1s. 6d. Index to Vol. XXVI., 2½d., post free.**

**The Monthly Part for February is now ready. Price 6d., by post 8d.**

### BOOKS RECEIVED.

J. and A. Churchill.

"Diet and Food." By Alexander Haig, M.A. and M.D.Oxon., F.R.C.P. Second edition.

Hazell, Watson, and Viney.

"The Mirage of Two Buried Cities." By John Fletcher Horne, M.D., F.R.S.E.

W. R. Russell and Co.

"Cookery for Two and More." By Ellis Peyton.

Kegan Paul, Trench, Trubner, and Co.

"Life and Happiness." By Auguste Marrot.

T. B. Browne.

"The Advertiser's A B C."

T. Fisher Unwin.

"Experiments on Animals." By Stephen Paget, with an introduction by Lord Lister.

### Scraps and Gleanings.

Her Majesty the Queen has forwarded 20 pheasants for the use of the patients in the Cancer Hospital, Brompton.

A successful subscription dance was given on January 26th in aid of the Withington Bed in the Ancoats Hospital.

The managers of the Edinburgh Infirmary have placed at the disposal of the War Office accommodation for 100 sick and wounded soldiers from South Africa, the majority to be placed in the Infirmary and a few in the convalescent home at Corstorphine.

At the first annual meeting of the Dundee Victoria Hospital it was stated that the amount originally secured for the erection and endowment of the hospital, viz., £50,000, was not proving sufficient for the needs. The income this amount brought in was £1,200 per annum, whilst £1,900 was required to maintain the hospital, which contains accommodation for 32 patients. Additional endowment contributions are therefore needed.

The subscriptions to the Glasgow Convalescent Home, Lenzie, during 1899, show an increase under all heads, with the exception of the amount received for ordinary subscribers, which shows a slight decrease as compared with 1898. The total subscriptions for 1899 amount to £1,885 odd, against £1,800 odd in 1898. The committee, however, have a debt of £1,017 to clear off, and they appeal to the public for increased support. The total number of patients admitted to the home during 1899 was 1,720, as against 1,689 in 1898.

The Extension Committee of the Edinburgh Royal Infirmary at the close of 1899 had the sum of £57,541 to their credit, and the estimated amounts required to complete works in progress or in contemplation amounted to £75,000, made up as follows: Balance on new pavilion for the diseases of women, £22,000; new ophthalmic pavilion, £30,000; new ear, throat, and nose pavilion, £18,000; additions to kitchen and stores, £5,000. It is hoped that the surplus of this total over the amount in hand, *i.e.*, about £17,500, will be provided by donations and legacies.

### The Student of Medicine.

### APPOINTMENTS.

B. E. G. Bailey, M.R.C.S., L.R.C.P., appointed Second House Physician to the City of London Hospital for Diseases of the Chest, Victoria Park. J. Barlow, M.D, F.R.C.S., appointed Additional Examiner in Clinical Surgery for the University of Edinburgh. A. B. Blair, M.B., appointed Medical Officer for the Banwell First District of the Axbridge Union. S. Fraser, M.B., appointed Medical Officer for the Edmonton District of the Edmonton Union. W. T. Hedley, M.B., C.M.-Edin., appointed Medical Officer for the Hallaton District of the Uppingham Union. A. A. Hope, M.B., appointed Medical Officer for the Fifth District of the Daventry Union. A. McCall, M.D.Glasg., appointed Surgeon to the Throat and Ear Department of the Boscombe Hospital, Bournemouth. G. McKie, M.B., appointed Assistant Medical Officer to the St. George's in-the-East Infirmary and Workhouse. A. S. Percival, M.B.Camb., appointed Honorary Surgeon to the Newcastle Eye Infirmary. W. H. Reed, M.R.C.S.Eng., L.S.A., appointed Medical Officer of Health to the Westbury Urban District Council.

Hospital of St. Francis.

M. Agar, M.R.C.S., L.R.C.P., appointed Surgeon in Charge of the Aural Department. F. J. Fielder, B.S., M.D., appointed Surgeon in Charge of the Ophthalmic Department. J. W. MacVine, M.B., C.M., appointed as Assistant Surgeon and Anæsthetist.

**THE UNIFORM SYSTEM OF ACCOUNTS, AUDIT, AND TENDERS, for Hospitals and Institutions, with Certain Checks upon Expenditure; also the Index of Classification.** Compiled by a Committee of Hospital Secretaries, and adopted by a General Meeting of the same, 18th January, 1892. And certain Tender and other Forms for securing Economy, By Sir HENRY BURDETT, K.C.B. Demy 8vo, profusely illustrated with Specimen Tables, Index of Classification, Forms of Tender, &c., cloth extra, 6s.

**ACCOUNT BOOKS FOR INSTITUTIONS, designed in accordance with the Uniform System of Accounts for Hospitals and Institutions.** By Sir Henry Burdett, K.C.B. Being a complete set of Account Books ruled in accordance with the Uniform System adopted by the Metropolitan Hospital Sunday Fund.

*(Full Description of Books and Prices sent post free on application.)*

London: The Scientific Press, Ltd., 28 & 29, Southampton Street, Strand, W.C.

Accouchement Requisites.

Ambulance Cases.

### NOW READY,

A REPRINT OF

### A NURSING HANDBOOK

TOGETHER WITH S. MAW, SON, AND THOMPSON'S

### COMPLETE CATALOGUE OF NURSING REQUISITES.

The above work is now republished at a cost of 1s. per copy. Messrs. S. M., S., and T. have been compelled to make this charge on account of the extreme popularity of the work and the great expense incurred in its production.

***Post Free on receipt of 1s.***

### S. MAW, SON, and THOMPSON,

7 to 12, ALDERSGATE STREET, LONDON, E.C.

